# *Sarcoptes scabiei* Induces Discrete NET Release, Ca^2+^ Fluxes and ROS Production Without Impairing Phagocytic Activity in Bovine Polymorphonuclear Neutrophils

**DOI:** 10.3390/ani16111628

**Published:** 2026-05-27

**Authors:** Camilo Larrazabal, Iván Conejeros, Daniela Grob, Sara López-Osorio, Anja Taubert, Carlos Hermosilla

**Affiliations:** 1 Institute of Parasitology, Biomedical Research Center Seltersberg (BFS), Justus Liebig University Giessen, 35392 Giessen, Germany; 2 Department of Veterinary Sciences and Public Health, Universidad Católica de Temuco, Temuco 4780000, Chile; 3 CIBAV Research Group, Veterinary Medicine School, Faculty of Agrarian Sciences, Universidad de Antioquia, Medellin 050010, Colombia

**Keywords:** *Sarcoptes scabiei*, mange, mites, NET formation, ROS, calcium

## Abstract

Sarcoptic mange is a skin disease caused by *Sarcoptes scabiei* infestations, characterized by dermatitis, pruritus, and exudative responses in both humans and animals. Biologically, the life cycle of *S. scabiei* is confined to the host’s skin (*stratum corneum*), where mite-derived molecules trigger the influx of innate immune cells, including polymorphonuclear neutrophils (PMN), which play a central role in skin inflammatory responses. The antimicrobial activity of PMNs is regulated by Ca^2+^ fluxes and includes the generation of reactive oxygen species (ROS), degranulation, and the release of neutrophil extracellular traps (NETs). NETs are web-like structures composed of chromatin and enzymes that can trap and eventually kill pathogens; however, their involvement in *S. scabiei* infestations in bovines remains unclear. Here, we investigated interactions between bovine PMN and *S. scabiei* mites, as well as PMN responses to *S. scabiei* antigen (*Sc*Ag). Functional parameters included NET release, Ca^2+^ fluxes, ROS production and phagocytic activity, determined by fluorescence microscopy, Fluo-4 staining, luminol-derived luminescence and flow cytometry, respectively. Current data show that *Sc*Ag, but not whole mites, induces a weak NET release in exposed bovine PMN. Additionally, *Sc*Ag drives rapid and sustained Ca^2+^ fluxes and ROS production over time, without altering the phagocytic capacity of PMN.

## Simple Summary

Sarcoptic mange is a contagious skin disease that affects humans and animals, caused by the mite *Sarcoptes scabiei*, which lives and reproduces within the outer layer of the skin. Infestation leads to intense pruritus, skin inflammation, and lesions that affect animal welfare and productivity. Overall, a mite-driven immune response includes the recruitment of immune cells like neutrophils. These cells can kill pathogens by producing toxic molecules, releasing antimicrobial substances, and forming web-like structures made of DNA and enzymes that may trap and kill microbes. However, little is known on how neutrophils from cattle respond to this ectoparasite. In this study, we examined how bovine neutrophils react when exposed either to whole mites or mite-derived antigen. We showed that mite antigen drove cellular activation, including calcium influxes and the production of reactive oxygen species. Mite antigen also induced a limited formation of neutrophil extracellular traps, while mites themselves hardly stimulated this response. These findings improve our understanding on how the bovine immune system reacts to sarcoptic mange and may contribute to the development of better strategies to control this disease in livestock.

## 1. Introduction

Members of the family Sarcoptidae are burrowing astigmatid mites parasitizing throughout their life and considered as stationary permanent ectoparasites. Sarcoptic mange, or scabiosis, is a zoonotic parasitic skin infestation that affects both humans and animals, caused by the acari species *Sarcoptes scabiei* [1]. Biologically, *S. scabiei* infestation is confined to host skin, where shortly after infestation, fertilized female mites burrow beneath the *stratum corneum*, accomplishing their nutritional needs to release eggs into epidermal tunnels by ingesting lysed host skin tissue and lymph, but causing a significant inflammatory host response [1]. Clinically, *S. scabiei* infestation in animals represents a highly contagious ectoparasitosis inducing dermatitis, severe pruritus and alopecia, leading to thickened, hyperkeratotic, and hyperpigmented skin, compromised feed efficiency and overall animal welfare impairment [2]. In domestic animals, sarcoptic mange may affect cattle, dogs, pigs, sheep and goats, but rarely cats and horses [1]. The preferred site of *S. scabiei* infestation depends on the host species, with mites being more frequently found on sparsely haired parts of the body, such as ears, face, muzzle or neck. Nevertheless, burrowing mites might spread all over the body of infested animals causing generalized scabiosis. As stated above, burrowing, feeding and defecation activities of *S. scabiei* mites cause intense pruritus, inflammation, hair loss and crust formation of dried exudate. Intense pruritus leads to excoriation, resulting in exudation and even hemorrhage of the affected skin surface. The cutaneous pro-inflammatory host innate response mainly reflects keratinocyte- and epithelial cell-derived reactions leading to leukocyte recruitment to the site of infestation [3,4]. In this context, given its location, *S. scabiei*-derived antigenic substances are present in surrounding tissue layers, thereby inducing an early host innate immune reaction, being characterized by cytokine/chemokine release promoting leukocyte migration [1,3,5,6,7].

During *S. scabei*-driven pro-inflammatory epidermal responses, polymorphonuclear neutrophils (PMN) infiltrate mite-infested tissue [1,4]. Infestation-driven PMN recruitment occurs rapidly [8,9,10,11], as described for both human and animal *S. scabei* infestation in vivo [3,4,12,13,14]. However, the role of PMN in *S. scabiei* infestations is still largely unknown. Overall, mammalian PMN own different effector mechanisms, such as phagocytosis, release of extracellular vesicles (EV), degranulation of antimicrobial peptides, release of danger-associated molecular patterns (DAMPs) and ROS production, which are tightly regulated by intracellular Ca^2+^ fluxes after PMN activation [15]. In terms of sarcoptic mange, studies on *S. scabiei*-derived proteins revealed that these mites can impair PMN phagocytosis, thereby promoting secondary infections and highlighting the relevance of PMN in the pathogenesis of clinically manifested scabiosis [16,17]. In addition, cumulated evidence has demonstrated the capability of PMN to release either nuclear or mitochondrial DNA as fine and delicate extracellular structures, designated as neutrophil extracellular traps (NETs) [8,9,11]. Although beneficial in the context of infectious diseases, excessive NET extrusion has also been implicated in pro-inflammatory disorders causing sterile inflammation and contributing to autoimmune skin diseases, such as *Lupus erythematosus* and psoriasis [18,19]. NETs are primarily composed of decondensed chromatin, being decorated by granular antimicrobial components, which include myeloperoxidase (MPO), neutrophil elastase (NE), lactoferrin, calprotectin, LL37, pentraxin, proteinase 3, and cathepsin G, among others [11,20]. This composition is particularly effective in terms of microorganism capture and killing [8]. Different NET phenotypes have been described: (i) diffuse NETs (*diff*NETs), (ii) spread NETs (*spr*NETs), and (iii) aggregated NETs (*agg*NETs) [21,22]. In detail, *diff*NETs show a spherical and compact shape and range between 25 and 28 µm diameter. *Spr*NETs are elongated web-like structures, characterized by a 15–17 nm diameter, distributed in elongated thin fibers. Finally, *agg*NETs are derived from many PMN converging in large NET clusters, measuring over 50 µm in diameter [21,22].

While NET formation can be induced by diverse bacterial and protozoan species, this effector mechanism also addresses larger metazoan parasites, which cannot be phagocytized [23,24,25]. Consistently, PMN cast NETs in response to parasitic nematode stages, e.g., from *Haemonchus contortus*, *Strongyloides stercoralis*, *Ostertagia ostertagi*, *Brugia malayi*, *Dirofilaria immitis* and *Angiostrongylus vasorum* [24,25,26,27,28,29]. Interestingly, trematodes like *Fasciola hepatica* or *Calicophoron daubneyi* are considered weak NET-inducers [22,30], suggesting that NET responses are differentially induced across metazoan species. In this context, so far, no evidence exists on NET release against parasitic mites in bovines. Thus, the aim of the current work was to characterize, for the first time, bovine PMN responses against *S. scabei* stages (eggs, nymphs, and adults) and specific antigens (*Sc*Ags).

## 2. Materials and Methods

### 2.1. Bovine PMN Isolation

Peripheral blood was collected from healthy adult dairy cows (*n* = 4) by jugular venipuncture into 30 mL sterile, heparinized tubes (Kabe Labortechnik, Nümbrecht, Germany). The blood was diluted 1:1 with sterile PBS supplemented with 0.02% EDTA (Sigma-Aldrich, St. Louis, MO, USA) and layered on 12 mL of Histopaque^®^-1077 (density  =  1.077 g/mL; Sigma-Aldrich). Samples were centrifuged at 800× *g* for 45 min at room temperature (RT). Following centrifugation, the plasma and buffy coat layers were carefully removed. Remaining red blood cells (RBCs) were resuspended in Hank’s Balanced Salt Solution (HBSS), and hypotonic lysis was performed by adding 1 volume of cold phosphate-buffered water containing 5.5 mM NaH_2_PO_4_ and 8.4 mM KH_2_PO_4_ (pH 7.2; all Merck, Darmstadt, Germany). After 1 min, isotonicity was restored by adding 2 volumes of hypertonic phosphate buffer [5.5 mM NaH_2_PO_4_, 0.46 M NaCl (pH 7.2; all Merck)]. Samples were then centrifuged at 600× *g* for 10 min at 20 °C. The resulting PMN pellet was washed three times with 1× HBSS and resuspended in 5 mL of 1× HBSS. Cell counts were performed using a Neubauer hemocytometer (Marienfeld, Lauda-Königshofen, Germany). Freshly isolated bovine PMN were allowed to rest for 30 min at 37 °C and 5% CO_2_ prior to experimental use.

### 2.2. Sarcoptes scabiei Isolation and Mite Antigen (ScAg) Preparation

*S. scabiei* mites were collected from clinically infested dairy cows farmed in Bavaria, Germany. Specifically, the affected area was carefully trimmed, and deep epidermal scrapings were performed via the use of a sterile sharp skin scraper to collect skin material containing mites. These samples were stored at 4 °C and transferred to the Institute of Parasitology, JLU Giessen, where samples were stored at 4 °C until final processing, in a time period between 1 and 3 months. Samples were transferred to conical tubes and treated with 6 mL of 10% potassium hydroxide (KOH; Merck) solution. Epidermal digestion was performed by heating in 15 s intervals until hair/skin structures were dissolved. The digested material was then centrifuged at 600× *g* for 3 min at RT. To concentrate *S. scabiei* stages, a flotation was performed using sugar solution (550 g sugar, 443 mL tap water, 7 mL formaldehyde 37%; density 1.3 g/mL; Merck). The samples were mixed with sugar solution, centrifuged at 600× *g* for 5 min at RT to concentrate the mites in the upper layer, which were then collected and washed three times with distilled water. Mite identity was confirmed microscopically following established morphological criteria (Deplazes et al., 2013) [31]. For preparation of *S. scabiei* antigen (*Sc*Ag), an undetermined number of concentrated mites were suspended in 300 µL sterile phosphate-buffered saline (PBS) and subjected to sonication using five cycles of 15 s in an ice bath (Sonorex RK31^®^ bath-type sonicator; Bandelin, Berlin, Germany). The suspension was centrifuged at 10,000× *g* for 20 min at 4 °C to separate insoluble debris. The protein concentration in the resulting supernatant was assessed by Bradford assay (Thermo Fisher Scientific, Waltham, MA USA) and aliquots were stored at −80 °C until further use as reported elsewhere [22].

### 2.3. Live Cell Analysis of Bovine PMN and Sarcoptes scabiei-Stage Interactions

Freshly isolated bovine PMN (2 × 10^5^ cells per sample; *n* = 3 different animals) were centrifuged at 300× *g* for 10 min at RT. Cells were then resuspended in 1 mL sterile RPMI 1640 medium without phenol red (Sigma-Aldrich) and loaded with Sytox Green (1:1000; Thermo Fisher Scientific) for extracellular DNA visualization. *S. scabiei* mites (nymphs and larvae) and eggs were placed in an 8 µ-Slide well (Ibidi, Gräfelfing, Germany) to adhere to the bottom. Subsequently, bovine PMN were added to mites and eggs in a total volume of 1 mL. Plates were incubated for 60 min at 37 °C and monitored microscopically by an inverted Olympus IX81^®^ fluorescence microscope (Olympus, Tokyo, Japan). Microscopic images were analyzed by ImageJ^®^ software (Fiji version 1.7, [32]) for quantification and visualization of extracellular DNA.

### 2.4. NET Visualization and Characterization of NET Phenotypes

Bovine PMN (*n* = 3; 2 × 10^5^ cells per sample) were stimulated with *Sc*Ag at concentrations ranging from 10 µg to 100 µg for 120 min at 37 °C in a 5% CO_2_ atmosphere. Briefly, cells were seeded on 15 mm diameter coverslips (Thermo Fisher Scientific) previously treated with 0.01% poly-_L_-lysine (Sigma-Aldrich) for 30 min in a total volume of 1 mL. Following stimulation with *Sc*Ag, PMN were fixed with 4% paraformaldehyde (Merck) and stored at 4 °C until further processing. To detect NET-specific proteins, samples were reacted with anti-histone (H1, H2A/H2B, H3, and H4; 1:200, Merck #MAB3422) and anti-neutrophil elastase (NE) (1:200, Abcam #ab68672, Cambridge, UK) antibodies as previously described [21]. Therefore, samples were first blocked for 60 min at RT with blocking buffer containing 3% bovine serum albumin (BSA; Sigma-Aldrich) and 0.3% Triton X-100 (Thermo Fisher Scientific). Then, primary antibodies were applied for 180 min at RT, followed by three washes with sterile PBS. Thereafter, secondary antibodies [Alexa Fluor 594 goat anti-mouse IgG (#A11005), Alexa Fluor 488 goat anti-rabbit IgG (#A11008), 1:500; Invitrogen, Carlsbad, CA, USA] were reacted for 30 min at RT in the dark. After three additional PBS washes, coverslips were mounted using Fluoromount G with DAPI (Thermo Fisher Scientific). Fluorescence imaging was performed using an inverted Olympus IX81 epifluorescence microscope equipped with an XM10 digital camera. Quantification of distinct NET phenotypes, i.e., spread NETs (*spr*NETs), diffuse NETs (*diff*NETs) and aggregated NETs (*agg*NETs), was performed by microscopic observation. NET phenotypes were identified and categorized based on morphological characteristics following established criteria [21,22].

### 2.5. Intracellular Ca^2+^ Flux Analysis and Live Cell 3D-Holotomographic Microscopy

Ca^2+^ fluxes in bovine PMN were assessed using the Ca^2+^-sensitive fluorescent dye Fluo-4-AM (Invitrogen), as described elsewhere (Conejeros et al., 2012) [33]. Briefly, bovine PMN were incubated with 2.5 µM Fluo-4-AM (Invitrogen) in 1× HBSS (30 min; 37 °C) using 5 × 10^7^ PMN/mL in a total volume of 1 mL. Excess dye was removed by washing the cells with 1× HBSS (Merck). Fluo-4-AM-loaded cells were then seeded in a 96-well plate at 5 × 10^6^ PMN/mL in a total volume of 200 µL. Experimental conditions included stimulation with *Sc*Ag (10 µg/mL) or A23187 (5 µM; Sigma-Aldrich). Ca^2+^ flux over time was quantified by area under the curve (AUC), using the first 50 s prior to stimulus exposure as baseline, and analyzing a total of 540 s in an automated multiplate reader (Varioskan Flash, Thermo Fisher Scientific). To confirm neutrophil Ca^2+^ fluxes, live cell 3D-holotomography coupled with epifluorescence was performed on Fluo-4-AM-loaded PMN stimulated with *Sc*Ag (10 µg/mL), as previously described [34]. Briefly, 1 × 10^5^ Fluo-4-AM-loaded PMN were seeded on 35 mm tissue culture µ-plates (Ibidi) and stimulated with *Sc*Ag (10 µg/mL) in a total volume of 1 mL. Live cell imaging was performed using a 3D Cell Explorer microscope (Nanolive, Tolochenaz, Switzerland) equipped with 60× magnification (λ  =  520 nm, sample exposure 0.2 mW/mm^2^, depth of field 30 µm) and a fluorescence unit (CoolLED pE-300 ultra). Images were acquired every 6 s in both refractive index (RI) and FITC fluorescence channels over 5 min. Raw data were processed using STEVE^®^ software v 1.6 (Nanolive) to generate RI-based z-stacks. Post-processing and analysis were performed with ImageJ. Average intensity-projected holotomographic z-stacks were generated using the Z Project plugin. Fluo-4-AM-based fluorescence images were shown using the “fire” pseudocolor scale to facilitate visualization of fluorescence intensity changes over time.

### 2.6. Quantification of Bovine PMN ROS Production

To evaluate the effects of *Sc*Ag stimulation on bovine PMN oxidative responses, a luminol-based chemiluminescence assay was performed, as previously described [35]. Briefly, 1 × 10^6^ PMN were suspended in sterile 1× HBSS and placed into a 96-well plate (Sarstedt, Leicester, UK) pre-warmed to 37 °C in a total volume of 200 µL. Next, 50 μM luminol (Sigma-Aldrich) was added and the samples gently mixed. Basal ROS production was recorded for 120 s prior to *Sc*Ag (10 µg/mL) supplementation, after which luminescence was continuously monitored for 1900 s using a Luminoskan microplate reader (Thermo Fisher Scientific).

### 2.7. Phagocytosis Assay

Heparinized bovine whole blood was incubated for 15 min at 37 °C in the presence or absence of *Sc*Ag (10 µg/mL). Then, the phagocytic activity of immune cells present in whole blood samples was assessed using pH-sensitive rhodamine-conjugated *Escherichia coli* (*E. coli*) particles (Invitrogen, Carlsbad, CA, USA). Briefly, pHrodo dye-labeled *E. coli* particles (20 µL) were added to 100 µL of *Sc*Ag-stimulated and non-stimulated whole blood samples. Samples were incubated for 15 min at either 37 °C or 4 °C (negative control for phagocytosis). Subsequently, red blood cells were lysed and the samples centrifuged (350× *g* for 5 min). The cell pellet was washed and PMN analyzed by flow cytometry (BD Accuri C6 Plus flow cytometer, BD Bioscience, Heidelberg, Germany). The bovine PMN population was gated based on forward and side scatter parameters. The phagocytosis rate was determined as percentage of pHrodo-positive PMN, using non-stained cells to set gating thresholds.

### 2.8. Statistical Analyses

Statistical analyses were performed via GraphPad Prism 8^®^ (version 8.4.3) software. Data description was carried out by presenting arithmetic means ± standard deviation. A Mann–Whitney test for the comparison of two experimental conditions was applied. In cases of three or more conditions, the Kruskal–Wallis test was used. Whenever global comparison by Kruskal–Wallis test indicated significance, post hoc multiple comparison tests were carried out by Dunn tests to compare test with control conditions. Outcomes of statistical tests were considered to indicate significant differences when *p*  ≤  0.05 (significance level).

## 3. Results

### 3.1. Sarcoptes scabiei Stages and ScAg Stimulation Barely Induce NETosis

**Figure 1:**
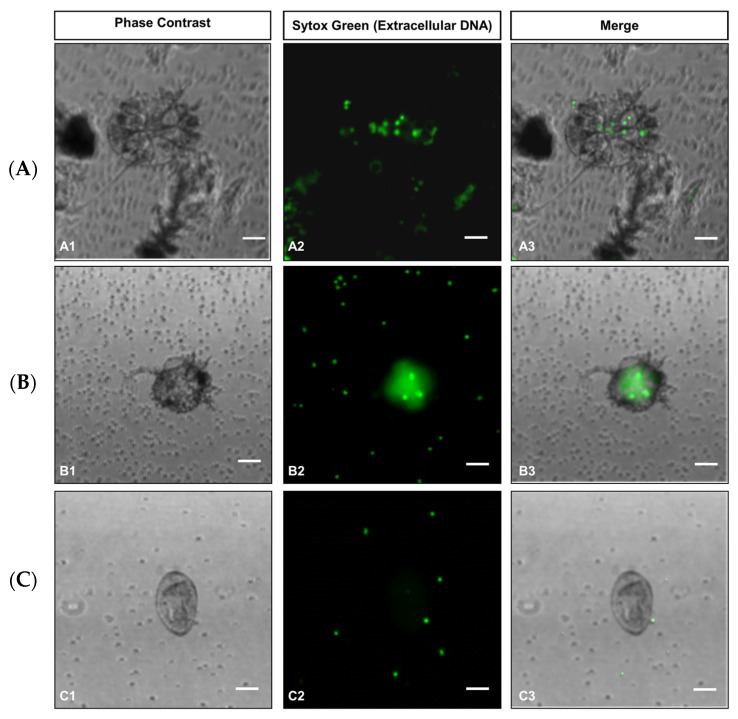
Extracellular DNA release by bovine PMN in response to intact *Sarcoptes scabiei* stages. Bovine PMN were co-cultured with *S. scabiei* nymphs (**A**), larvae (**B**), or eggs (**C**) in medium containing Sytox Green to visualize extracellular DNA release. Images show phase contrast (**1**), fluorescence (**2**), and merged (**3**) views. Scale bar: 100 µm.

**Figure 2:**
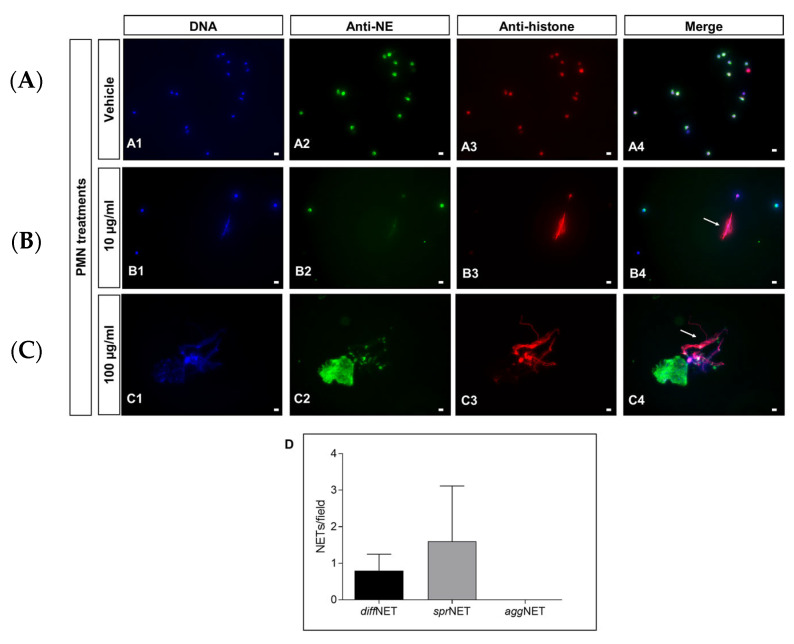
Immunofluorescence analysis of NET formation induced by *Sarcoptes scabiei*-derived antigen. Bovine PMN were incubated with vehicle control (**A**) or stimulated with *S. scabiei* antigen (*Sc*Ag) at 10 µg/mL (**B**) or 100 µg/mL (**C**). (**D**) Bar graph illustrating mean ± SD of different NET phenotypes per field from *Sc*Ag (10 µg/mL)-treated cells. Immunofluorescence images show NET-like structures composed of 1: DNA (blue), 2: neutrophil elastase (green) and 3: histones (red) or 4: merge. Arrow indicates NET-like structures induced by *Sc*Ag treatment. Scale bar: 5 µm.

To evaluate if different stages of *S. scabiei* (i.e., eggs, larvae and nymphs) drive NET release in bovine PMN, DNA release was first assessed by Sytox Green-derived epifluorescence in live cells. This approach allowed for the visualization of extracellular DNA release in PMN–mite co-cultures. As shown in Figure 1, bovine PMN attached to *S. scabiei* nymphs (Figure 1(A3)) and larvae (Figure 1(B3)), maintaining their characteristic round morphology. However, no extracellular DNA filaments indicative of NET extrusion were clearly observed after 1 h of co-culture with nymphs (Figure 1A), larvae (Figure 1B), or eggs (Figure 1C), regardless of cellular attachment. Notably, NET structures were not evident even in PMN attached to nymphs (Figure 1(A1–A3)) or eggs (Figure 1(C1–C3)), while only a minor PMN enlargement and diffuse Sytox green-derived signal were observed on PMN attached to larvae (Figure 1(B1–B3)), suggesting that physical interaction alone does not trigger NETosis. To further assess the NET-inducing capacity of *S. scabiei*, *Sc*Ag was used at 10 and 100 µg/mL to stimulate bovine PMN. Immunofluorescence analysis was employed to identify classical NET components: extracellular DNA, NE, and histones. As shown in Figure 2, stimulation of PMN with 10 µg/mL *Sc*Ag induced a modest NET response after 2 h, characterized by short, thread-like NET structures (*spr*NETs; Figure 2(B1–B4)). Co-localization of extracellular DNA (blue, Figure 2(B1)), NE (green, Figure 2(B2)), and histones (red, Figure 2(B3)) confirmed the presence of *Sc*Ag-induced NETs (Figure 2(B4)). In contrast, vehicle-treated control cells exclusively showed an intracellular localization of these components without NET formation (Figure 2(A1–A4)). In addition, a higher *Sc*Ag concentration (100 µg/mL) triggered cytotoxic responses in stimulated PMN (Figure 2(C1–C4)), as evidenced by disorganized co-localization of NET markers and absence of intact, non-NETotic cells. Phenotypic analyses of NETs, based on previously reported classification criteria [21], revealed that *spr*NETs represented the predominant phenotype observed in *Sc*Ag-stimulated cells at 2 h post-treatment, followed by *diff*NETs. Overall, *agg*NETs were not detected after mite stimulation (Figure 2D).

### 3.2. ScAg Induces a Fast and Sustained Ca^2+^ Flux in Bovine PMN

**Figure 3:**
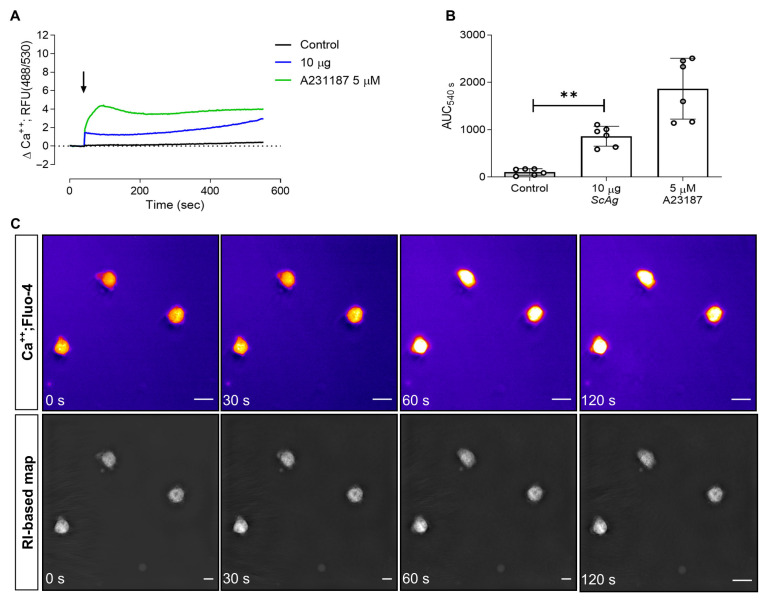
Stimulation with *Sarcoptes scabiei* antigen induces a rapid and sustained calcium flux in bovine PMN. Bovine PMN were loaded with Fluo-4 AM in HBSS/Ca^2+^ buffer and stimulated with *Sc*Ag (10 µg/mL) or the calcium ionophore A23187 (5 µM). (**A**) Representative trace shows intracellular Ca^2+^ kinetics over time. Arrow indicates stimulus addition (**B**) Bar graph represents the AUC at 540 s. (**C**) Time-lapse images of Fluo-4-loaded PMN are shown as pseudocolor and refractive index (RI)-based intensity maps following stimulation. Data represents the mean ± standard deviation of six biological replicates. ** *p* ≤ 0.01.

To determine whether *Sc*Ag triggers early activation of bovine PMN, intracellular Ca^2+^ fluxes were assessed as previously described [33]. As shown in Figure 3A, stimulation with 10 µg/mL *Sc*Ag induced a rapid and sustained increase in Ca^2+^-based fluorescence, even though the peak intensity was lower than that induced by the positive control A23187 (5 µM; 2.6 µg/mL). Kinetic analyses revealed that *Sc*Ag-induced Ca^2+^ mobilization in PMN remained consistent over time, with an increase in slope approximately 250 s after stimulation. A quantitative evaluation of the data via AUC analysis confirmed that stimulation with *Sc*Ag significantly elevated intracellular Ca^2+^ levels in PMN over the measurement period (*p* = 0.002), with the mean signal being approximately 2.2-fold lower than that generated by A23187 treatments (Figure 3B). These findings were supported by live cell 3D holotomographic fluorescence microscopy (Figure 3C), which confirmed a sustained increase in cytoplasmic Ca^2+^ signals following neutrophil *Sc*Ag stimulation. Notably, this activation mode occurred without phenotypic changes in PMN throughout the recording period.

### 3.3. ScAg Induces Oxidative Responses in Bovine PMN

**Figure 4:**
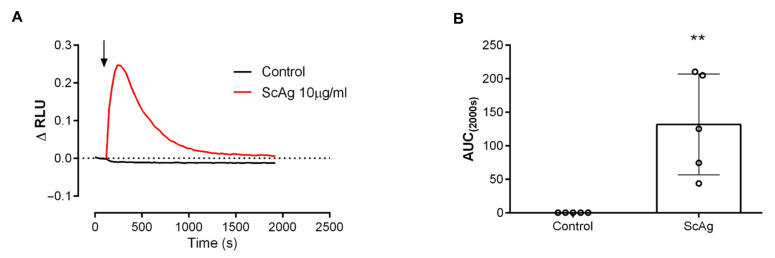
Stimulation with *Sarcoptes scabiei* antigen induces ROS production in bovine PMN. ROS generation was evaluated by luminol-based chemiluminescence. (**A**) Representative luminometric traces over time of PMN stimulated with *Sc*Ag (10 µg/mL) or vehicle. Arrow indicates stimulus addition (**B**) Bar graph shows the AUC at 2000 s. Data represents the mean ± SD of six biological replicates. ** *p* ≤ 0.01.

Since *ScAg* was shown to induce NET formation and intracellular Ca^2+^ fluxes in bovine PMN, we next assessed neutrophil oxidative responses driven by this mite-derived antigen using luminol-based chemiluminescence assays, as previously described [35]. Overall, stimulation with 10 μg/mL *Sc*Ag elicited a rapid and sustained increase in ROS-based luminescence, peaking approximately 240 s after exposure. This response gradually declined, returning to baseline levels around 900–1000 s post-stimulation (Figure 4A). Quantitative AUC analysis confirmed a significant elevation of ROS production over time compared to vehicle-treated controls (*p* = 0.0079) (Figure 4B).

### 3.4. ScAg Does Not Affect Bovine PMN Phagocytosis

**Figure 5:**
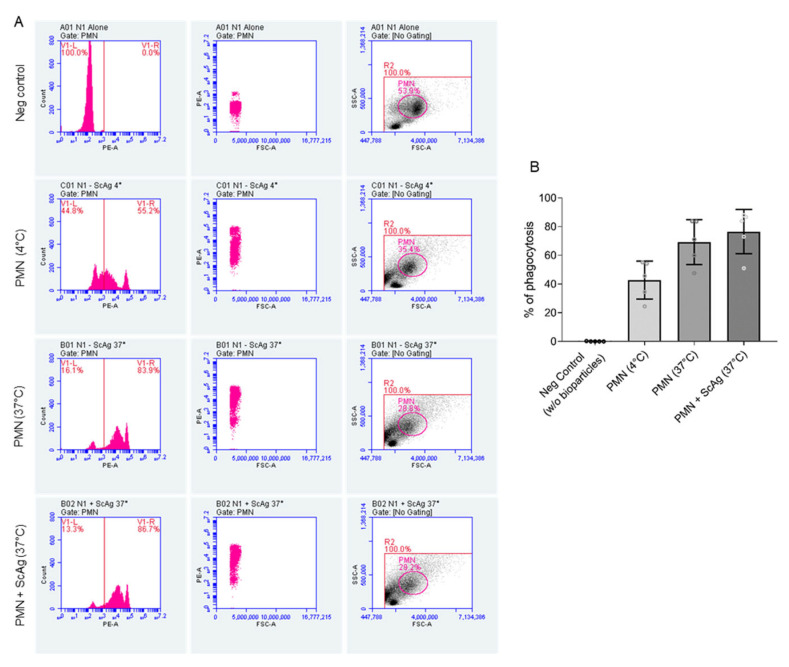
PMN-derived phagocytosis of pHrodo–*E. coli* bioparticles is not affected by stimulation with *Sarcoptes scabiei* antigen. PMN were exposed to *E. coli* bioparticles conjugated with the pH-sensitive probe pHrodo. (**A**) Representative histograms and forward/scatter dot plots illustrating the gating strategy for untreated and *Sc*Ag-exposed PMN. (**B**) Bar graph representing the mean percentage of phagocytosis ± SD of five biological replicates.

Based on previous reports indicating that *S. scabiei*-derived molecules can modulate PMN phagocytic activity [17], we further studied if stimulation with *Sc*Ag affects the phagocytic activity of bovine PMN. Phagocytosis was assessed by flow cytometry using pHrodo-labeled bioparticles, a reliable indicator for this defense function. As shown in Figure 5, an average of 69.2 ± 15.64% of PMN were engaged in phagocytosis under baseline conditions. Importantly, pre-treatment with *Sc*Ag did not significantly alter this percentage, with 76.52 ± 15.41% of PMN performing phagocytosis, indicating no observable effect of *Sc*Ag on neutrophil phagocytic activity.

## 4. Discussion

Sarcoptic mange is a globally distributed infectious skin disease of mammals caused by *S. scabiei* infestation. Due to the parasite’s life cycle, close interactions between its developmental stages and components of the host’s innate immune cells are very likely during infestation [1,4]. Similar to other cutaneous inflammatory responses, sarcoptic mange is associated with endothelial cell activation, increased vascular permeability, and rapid infiltration of immune cells like PMN [12,13,36,37,38,39]; however, the specific role of NET formation and other neutrophil effector mechanisms during this parasitic infestation remains largely unexplored. In this study, we examined interactions between bovine PMN and different *S. scabiei* stages in vitro. Co-culture of bovine PMN with *S. scabiei* eggs, larvae and nymphs revealed minimal PMN attachment to the parasite’s chitinous exoskeleton, suggesting that PMN are not effectively attracted by mites and fail to directly interact with the parasitic surface. The latter effect could be a consequence of the KOH-based mite isolation method, where mite surface proteins can potentially be damaged. However, this finding is consistent with previous reports showing that PMN tend to accumulate in perivascular regions of the dermis during *S. scabiei* infestation rather than directly interacting with mite structures [13]. The limited chemotaxis and/or attachment suggests that surface antigenic components of the mites may not represent adequate triggers of PMN engagement. Similar findings were reported in *F. hepatica* infections, where juvenile flukes show limited interactions with host PMN [22], supporting the idea that some metazoan parasite stages may be capable of evading initial PMN responses. The same holds true for the ruminant amphistome species *C. daubneyi,* where different parasitic stages and parasite-specific antigens (*Cd*Ag) merely induced discrete NET formation in exposed bovine PMN [30].

Mammalian PMN rely on several effector mechanisms to combat pathogen invasion, including phagocytosis, degranulation, oxidative burst, EV and NET release [9,40,41]. Obviously, during metazoan infections, phagocytosis proves to be ineffective due to the large size of the pathogen; thus, alternative responses like NETosis come into play [24,25,26,27,29]. In the current study, PMN co-cultures with *S. scabiei* stages failed to induce considerable NET formation in bovine PMN, indicating a low level of PMN activation in response to the parasite exoskeleton encounter. PMN, like other innate immune cells, rely on pattern recognition receptors (PPRs), such as TLRs and Dectin-1, to recognize pathogen-associated molecular patterns (PAMPs) and to initiate immune responses [42,43]. To test whether *S. scabiei*-derived molecules can activate PMN independent of physical contact, we stimulated PMN with soluble *Sc*Ag. In contrast to intact parasite stages, stimulation with 10 µg/mL of *Sc*Ag triggered moderate NET formation in bovine PMN. NET identity was confirmed by the co-localization of NE, histones, and extracellular DNA, and the predominant phenotype observed was short *spr*NETs. This discrete NETotic response is consistent with previous findings in other parasite stages like juvenile *F. hepatica* [22] and adult *C. daubneyi* [30]. Current results confirm that the magnitude and phenotype of NET responses may be strongly influenced by specific immunogenic properties of individual parasite stages and species, as previously proposed [22,30].

Ca^2+^ flux-based signaling is a central regulator of PMN effector functions, including degranulation, ROS generation and NET formation [15,44,45]. Overall, we here assessed whether stimulation with *Sc*Ag affects intracellular Ca^2+^ homeostasis in bovine PMN. Current data demonstrated that *Sc*Ag stimulation induces a rapid and sustained increase in neutrophil intracellular Ca^2+^ levels, indicating early PMN activation upon antigen exposure. In PMN, Ca^2+^ flux is typically induced by chemoattractants like CXCL8, PAF, or LTB4, as well as by interactions with adhesion molecules (L-selectin or CD11b) and Fc receptors [46]. These triggers of Ca^2+^ signaling usually precede downstream functional PMN responses. However, in the context of metazoan-derived molecules, including parasitic arthropods, the mechanisms underlying PMN activation remain less defined. For instance, excretory/secretory products of the blowfly *Lucilia sericata* (Calliphoridae) have been shown to elicit both oxidative and non-oxidative PMN responses via Ca^2+^-independent pathways [47], suggesting that helminth- or arthropod-derived molecules may activate mammalian PMN through non-canonical or PRR-independent signaling routes. In line with this, our findings indicate that *S. scabiei*-derived antigens can act as PMN modulators via Ca^2+^-dependent signaling but in a rather moderate way. Further studies should address the participation of different Ca^2+^-linked mechanisms, such as intra- and extra-cellular Ca^2+^ origin and store-operated Ca^2+^ entry (SOCE), upon *S. scabei*-induced PMN activation in bovines.

Since NET formation is classically linked to oxidative responses [9], we next evaluated the capacity of *Sc*Ag to induce neutrophil ROS production. Current results show that *Sc*Ag stimulation triggers a rapid and sustained generation of ROS in neutrophils, confirming its potential as an oxidative stimulus for bovine PMN. In PMN, ROS production is primarily mediated by NADPH oxidase (NOX) activation [9], catalyzing the reduction of molecular oxygen (O_2_) to superoxide anion (O_2_^−^), which is subsequently converted to hydrogen peroxide (H_2_O_2_) and hypochlorous acid (HOCl) [45]. These processes create an oxidative microenvironment that mediates pathogen killing and supports NET formation. To our best knowledge, this is the first report on ROS production by PMN in response to *S. scabiei*-derived antigens, thus expanding the current understanding of arthropod-induced innate immune responses in bovines. This provides a novel mechanism in which PMN contribute to *S. scabiei*-induced inflammatory responses and tissue damage in bovines upon *S. scabiei* infestation.

During *S. scabiei* infestation *in vivo*, parasite-driven immunomodulation is thought to delay local host immune responses, thereby allowing a persistent (chronic) infestation [1,4]. In cases of scabiosis, secondary colonization of the epidermis by opportunistic bacteria like *Staphylococcus aureus* is frequently observed [4,12,14], furthermore indicating a compromised PMN-dependent defense. In this context, to assess a functional impact of *Sc*Ag on bovine PMN, we evaluated neutrophil phagocytic activities following *Sc*Ag stimulation. Current data indicate that *Sc*Ag, at concentrations sufficient to activate PMN oxidative and calcium responses, does not significantly alter the phagocytic activity of bovine PMN. This observation contrasts with previous findings reporting that recombinant SMSB4, a serpin derived from *S. scabiei*, can inhibit complement activity and reduce PMN phagocytosis [17]. Interestingly, the immunomodulatory function of serpins has also been documented in other parasitic arachnids, including the hard tick species *Amblyomma americanum*, *Ixodes ricinus*, and *Rhipicephalus microplus*, where salivary gland-derived serpins facilitate immune evasion and prolonged feeding times by dampening host immune responses [48,49,50]. In this context, it is plausible to hypothesize that either the current antigens derived from whole specimens contained amounts of serpins that were too low to induce suppressive effects or that the modulatory effects of *S. scabiei-*derived serpins on phagocytosis are primarily exerted through complement-dependent routes, which was not studied in the current experimental approach. However, these assumptions need further investigations for a final conclusion.

## 5. Conclusions

The current data indicate that *S. scabiei* antigens drive different events of early activation in bovine PMN, including intracellular Ca^2+^ flux and ROS production, and induce a modest release of NETs. However, these innate immune responses are not linked to a change in phagocytic activity. Notably, co-cultures with intact *S. scabiei* eggs, larvae and nymphs failed to activate PMN or to promote significant PMN chemotaxis or cell attachment, suggesting that the parasite’s exoskeleton may lack accessible antigens or immunostimulatory surface components recognizable by bovine PMN during early infestation. Despite that, since *Sc*Ag induced ROS production and Ca^2+^ fluxes in bovine PMN, the participation of PMN-derived responses during bovine scabiosis could enhance inflammatory responses, increasing mite-induced tissular damage or hampering tissular reparation. Nonetheless, given the exploratory design and the limited number of animals included in this study, these finding should be carefully interpreted. Further studies with larger samples sizes are required to fully exclude individual variability.

## Data Availability

All data are included in the manuscript.
